# Consumer purchasing patterns in response to calorie labeling legislation in New York City

**DOI:** 10.1186/1479-5868-8-51

**Published:** 2011-05-27

**Authors:** Maya K Vadiveloo, L Beth Dixon, Brian Elbel

**Affiliations:** 1 Department of Nutrition, Food Studies and Public Health, New York University, NY 35 West 4th St NY, NY 10012 USA; 2 Department of Nutrition, Food Studies and Public Health, New York University, NY 35 West 4th St NY, NY 10012 USA; 3 NYU School of Medicine and NYU Wagner School of Public Service, 423 E. 23rd Street, 15-120N, NY, NY 10010 USA

## Abstract

**Background:**

Obesity is a major public health threat and policies aimed at curbing this epidemic are emerging. National calorie labeling legislation is forthcoming and requires rigorous evaluation to examine its impact on consumers. The purpose of this study was to examine whether point-of-purchase calorie labels in New York City (NYC) chain restaurants affected food purchasing patterns in a sample of lower income adults in NYC and Newark, NJ.

**Methods:**

This study utilized a difference-in-difference design to survey 1,170 adult patrons of four popular chain restaurants in NYC and Newark, NJ (which did not introduce labeling) before and after calorie labeling was implemented in NYC. Receipt data were collected and analyzed to examine food and beverage purchases and frequency of fast food consumption. Descriptive statistics were generated, and linear and logistic regression, difference-in-difference analysis, and predicted probabilities were used to analyze the data.

**Results:**

A difference-in-difference analysis revealed no significant favorable differences and some unfavorable differences in food purchasing patterns and frequency of fast food consumption between adult patrons of fast food restaurants in NYC and Newark, NJ. Adults in NYC who reported noticing and using the calorie labels consumed fast food less frequently compared to adults who did not notice the labels (4.9 vs. 6.6 meals per week, p <0.05).

**Conclusion:**

While no favorable differences in purchasing as a result of labeling were noted, self-reported use of calorie labels was associated with some favorable behavioral patterns in a subset of adults in NYC. However, overall impact of the legislation may be limited. More research is needed to understand the most effective way to deliver calorie information to consumers.

## Background

Overweight and obesity and associated comorbidities are major contributors to avoidable mortality in the United States [1]. Nationwide, nearly 70% of adults are categorized as either overweight or obese [2]. The economic burden of this disease has led to consideration of environmental factors that could favorably affect disease prevention and progression. In 2008, New York City (NYC) became the first city to enact mandatory calorie labeling legislation as a public health strategy intended to inform consumers' away from home food purchases [3]. The present legislation requires restaurant chains with 15 or more outlets nationally to clearly post the calorie content of regular menu items next to the price on menu boards at the point-of-purchase [3]. Similar federal legislation is awaiting implementation as part of the Patient Protection and Affordable Care Act [4].

Preliminary analyses of the efficacy of this policy and of interventions targeting the provision of calorie information at the point-of-purchase (POP) have been mixed [5-12]. Individual-level factors such as gender [7] as well as contextual factors such as educational messages regarding recommended daily calorie intake [13] may moderate the efficacy of this policy. Moreover, calorie labels may result in healthier purchases for some consumers more than others. Some evidence suggests that adults are more willing to make healthier purchases for their children rather than for themselves [10]. However, a recent study observed no effect of parental involvement on healthier purchases at fast food outlets in NYC [14]. Five of six reviewed studies revealed some effect of POP labeling on food choices within a cafeteria or restaurant setting [7]. In one of these studies, sales data revealed that patrons reduced purchases of carbohydrate-rich foods, full-fat dairy products, and red meat [15]. Another study noted reductions in total calories purchased in a university cafeteria setting without any reduction in total sales volume [16].

Previously, Elbel et al [17] examined the effects of calorie labeling legislation before and 4-weeks after it was enacted in NYC, and did not find any significant differences in the total number of calories purchased by low-income adults in NYC and Newark, NJ (utilized as a comparison city). However, adults may have had other behavioral responses to calorie labeling legislation, such as reducing the frequency of fast food consumption or choosing calorically equivalent, but more nutritious foods and beverages. For example, adults may have opted to reduce their purchase of dessert items while simultaneously increasing their purchase of salads and salad dressing, which can be similarly high in calories, but with greater quantities of vitamins and minerals. The purpose of this study was to examine the data collected by Elbel et al to determine whether differences in food purchases or frequency of fast food consumption were observed in lower income adults in NYC and Newark, NJ.

## Methods

### Overview

The selection process for the cities, neighborhoods, restaurants, and survey procedures has been previously described [17]. Independent cross-sectional samples of adults were selected from NYC and Newark, NJ before and after calorie labeling legislation was enacted in NYC beginning on July 8, 2008. Newark, NJ was selected as the comparison city because of its similar demographic characteristics to NYC, but lack of calorie labeling legislation. According to data from the 2000 US Census [18], approximately 28% of residents in Newark, NJ and 20-38% of residents in the surveyed boroughs of NYC (Bronx, Brooklyn, Manhattan, Queens) were living at or below the federal poverty level. Restaurants in NYC and Newark, NJ were matched based on six population characteristics including population size, age, race/ethnicity, poverty level, and obesity and diabetes prevalence. The selected chain restaurants (McDonalds, Burger King, Wendy's, Kentucky Fried Chicken) were located in lower-income areas with a higher proportion of African-American and Latino minority residents ranging from 46% to 91% African-American and Latino. A total of five restaurants in Newark, NJ and 14 restaurants in NYC were sampled.

Patrons of the selected restaurants located in NYC and Newark, NJ were asked to participate in a survey prior to the implementation of calorie labeling legislation in NYC. Separate samples of patrons at each location were asked (at the same time of day and day of week) to participate in the same survey 4-weeks after legislation was implemented in NYC. Using a technique similar to a "street-intercept" survey [19], patrons were approached as they entered the selected restaurants on Tuesday through Thursday during the lunch (12:30 - 3:00 p.m.) or dinner (4:30 - 7:00 p.m.) hours in order to best represent "usual" vs. "special occasion" eating habits. Patrons who agreed to participate provided study researchers with their receipt and responded to a short survey. During the pre-labeling and post-labeling periods, patrons were queried about their food purchases, any alterations made to those purchases (e.g., added cheese, type of soda), and whether they shared the meal with anyone. Information about participants' age, race/ethnicity, and gender was collected. During the post-labeling period, patrons were queried about the degree to which they noticed and used the calorie labels to make food purchases. They were also asked about their formal education.

The New York University School of Medicine Institutional Review Board approved the study protocol.

### Population and Eligibility Criteria

Receipts and surveys were gathered from 1,396 individuals. For this study, only 1,170 adults aged 18 years and older were included in the analytic sample. During the pre-labeling period, 384 adults in NYC and 182 adults in Newark, NJ were surveyed; 442 adults in NYC and 162 adults in Newark, NJ were surveyed during the post-labeling period. Additionally, a subgroup of 440 adults in NYC during the post-labeling period were analyzed separately to determine whether the degree to which patrons noticed and reported using the calorie information was related to food purchasing patterns. Two adults were excluded because they did not respond to these questions.

### Outcome Measures

The following types of food purchases were compared in this study because they were considered feasible changes that adult patrons may have made to their fast food consumption after noticing calorie labels:

Type of beverage purchased: whether purchased beverage was caloric (e.g., soda, juice, milk) versus non-caloric (e.g., water or diet soda).

Salads purchased: whether a green salad was ordered at each establishment.

Type of salad dressing purchased: whether the salad dressing was full fat (i.e., regular) or reduced in calories. Adults who ordered a salad but did not select any salad dressing were combined with adults who ordered reduced fat dressings.

French fries purchased: whether French fries of any size were ordered either as part of a meal combo or as a separate item.

Addition of cheese to menu items: whether cheese was selected as an "add on" to menu items (e.g. hamburgers and sandwiches).

Number of desserts ordered: whether a dessert was ordered, particularly milkshakes or ice cream orders.

Frequency of fast food consumption per week: total number of breakfast, lunch, dinner, snacks, and total meals (the sum of these) consumed at fast food restaurants as reported by each adult.

### Statistical methods

Statistical analyses were performed using Stata 11.1 (StataCorp LP College Station, Texas). In addition to descriptive statistics, chi-square tests were run to compare differences between adults in NYC and Newark, NJ pre- and post-labeling, and within NYC and Newark, NJ pre- and post-labeling. A difference-in-difference analysis was used to examine whether differences in adults' food and beverage purchases and frequency of fast food consumption pre- and post-labeling in NYC were significant above the pre- and post-labeling differences in Newark, NJ. The intention of this type of analysis is to ensure that observed differences between groups result from a specific intervention (i.e., calorie labels) rather than other changes that may have occurred during the same time period. Linear and logistic regression analyses were run to examine whether participants' self report of whether they noticed and used calorie information affected food purchases of adults in NYC during the post-labeling period. Comparisons were made between adults who reported not noticing the information, noticing the information but not using the information, and noticing and using the information to make food purchasing decisions. All analyses used clustered standard errors at the restaurant level to account for correlation between adults sampled from the same restaurant. *P*-values less than 0.05 were considered statistically significant.

## Results

Table 1 presents the demographic and descriptive characteristics of the study samples in NYC and Newark, NJ before and after the implementation of calorie labeling legislation in NYC. Prior to the passage of calorie labeling legislation, the race/ethnicity composition of the study samples differed significantly between NYC and Newark, NJ. This difference remained significant post-legislation. The type of fast food restaurants where adults agreed to participate also differed significantly pre- and post-labeling in NYC and Newark and also differed within NYC pre- vs. post-labeling. However, overall at least 10% of adults came from every type of restaurant, and both race and restaurant type were included as covariates in all analyses. Age and gender were also included as covariates in the final model.

**Table 1:** Demographic and Descriptive Characteristics of Adult Patrons at Fast Food Restaurants in NYC and Newark, NJ

	New York City		Newark, NJ		Significance			
	Before Labeling (n = 384)	After Labeling (n = 442)	Before Labeling (n = 182)	After Labeling (n = 162)	Pre-labeling	Post-labeling	Pre-post NYC	Pre-post Newark
**Mean Age (std. error)**	39.1 (0.74)	38.8 (0.68)	40.4 (0.90)	37.7 (.97)	*			
**Race/Ethnicity (%)**					***	***		
White	9.9	6.8	6.0	4.9				
Black	57.0	63.0	74.2	81.5				
Latino	25.5	21.6	14.3	9.3				
Asian/Hawaiian Pacific Islander	2.3	1.6	1.7	1.2				
Other	4.4	5.9	1.7	0.6				
**Females (%)**	61.8	65.1	59.8	59.0				
**Observer-Estimated Percent Obese (based on estimated BMI)**	9.1	8.1	8.2	7.3				
**Restaurant Chain (%)**					***	***	***	
McDonalds	45.6	34.4	25.4	29.6				
Burger King	11.9	12.0	31.2	30.3				
Wendy's	25.6	36.0	20.8	15.4				
Kentucky Fried Chicken	17.0	17.7	22.5	24.7				

*p <0.10; **p<0.05; ***p<0.01;

¶ test of significance between NYC v. Newark pre-labeling; NYC v. Newark post-labeling; pre v. post labeling in NYC; pre v. post labeling in Newark

Table 2 presents differences in food and beverage purchases and frequency of fast food consumption before and after calorie labeling in NYC and Newark, NJ. At baseline, the percentage of adults who ordered a caloric beverage was significantly greater in Newark, NJ (37%) compared to NYC (12%) (p <0.01). After labeling, the percentage of adults who ordered a caloric beverage increased significantly in NYC (12% vs. 18%, p <0.05), and the difference between NYC and Newark, NJ was no longer significant. However, the difference-in-difference results between NYC and Newark, NJ were significant (p <0.05). While no significant differences existed between NYC and Newark, NJ at baseline with respect to number of salads ordered, there was a significant difference in number of salads ordered after calorie labeling in both cities, with a greater percentage of adults ordering a salad in NYC (8%) than in Newark, NJ (3%) (p <0.05). However, the difference-in-difference analysis was not significant. At baseline, a smaller percentage of NYC adults ordered regular salad dressing (12% vs. 40%, p <0.05). This percentage significantly increased in NYC after labeling (39%), and the difference-in-difference results between NYC and Newark, NJ were significant (p <0.01). However, only a few adults ordered salads, as noted above.

**Table 2:** Differences in Beverage and Food Consumption between Adult Patrons at Fast Food Restaurants in NYC and Newark, NJ

	New York City		Newark, NJ		Significance				
	Before Labeling (n = 384)	After Labeling (n = 442)	Before Labeling (n = 182)	After Labeling (n = 162)	Pre-labeling	Post-labeling	Pre-post NYC	Pre-post Newark	Difference-in-Difference†
**Caloric Beverage (yes)**	47/384 = 12%	81/442 = 18%	67/182 = 37%	23/162 = 14%	***		**	***	**
**Ordered a Salad (yes)**	50/384 = 13%	36/442 = 8%	15/182 = 8%	5/162 = 3%	*	**	**	**	
**Type of Salad Dressing (regular)**	6/50 = 12%	14/36 = 39%	6/15 = 40%	1/5 = 20%	**		***		***
**Orders of French Fries**	121/384 = 32%	162/442 = 37%	58/182 = 32%	50/162 = 31%					
**Use of Cheese**	131/251 = 52%	142/262 = 54%	64/115 = 56%	61/85 = 72%		***		**	
**Dessert (yes)**	67/384 = 17%	59/442 = 13%	30/182 = 16%	15/162 = 9%			*	**	
**Mean Number of Fast Food Breakfasts per Week**	1.21	1.28	1.69	1.49		*			
**Mean Number of Fast Food Lunches per Week**	2.30	2.33	2.69	2.49	*	*			
**Mean Number of Fast Food Dinners per Week**	1.29	1.09	1.58	1.28		**	*		
**Mean Number of Snacks per Week**	1.31	1.19	1.29	1.22		***			
**Mean Number of Fast Food Meals per Week (including snacks)**	6.1	5.8	7.3	6.4	*	*			

*p≤0.10; **p <0.05; ***p <0.01;

¶ test of significance between NYC v. Newark pre-labeling; NYC v. Newark post-labeling; pre v. post labeling in NYC; pre v. post labeling in Newark

† Difference-in-difference analysis controlling for age, race, gender, and restaurant type and standard errors were clustered at the restaurant level

At baseline, there were no significant differences between NYC and Newark, NJ with regard to use of cheese on hamburgers. After labeling, however, there were significant differences in the use of cheese on hamburgers between NYC and Newark, NJ (54% vs. 72%, p <0.01), but the difference-in-difference result was not significant. At baseline, the number of fast food dinners and snacks per week did not differ significantly between NYC and Newark, NJ. After labeling, the frequencies were reduced in NYC compared to Newark, NJ (1.09 vs. 1.28 dinners, p <0.05; 1.19 vs. 1.22 snacks, p <0.01), but the difference-in-difference analysis was not significant. No significant differences were found for orders of French fries, frequency of fast food breakfast or lunch, and frequency of overall meal consumption.

Table 3 presents the characteristics of adults in NYC in the post-labeling period who had an opportunity to notice and use the calorie information. The three groups of adults were similar with the exception of age; adults who did not notice the labels were significantly older than adults who did notice the labels and did or did not use the information.

**Table 3:** Demographic Characteristics of Adult Patrons in NYC after Menu Labeling Legislation

	Did Not See Calorie Information (Reference Group) (n = 196)	Saw Calorie Information But Did Not Use Information (n = 180)	Saw Calorie Information and Did Use Information (n = 64)	Significance
**Mean Age (std. error)**	41.1 (1.10)	36.6 (0.97)	38.0 (1.77)	***
**Race/Ethnicity (%)**				
White	6.6	5.6	10.9	
Black	65.3	63.3	54.7	
Latino	21.9	22.8	17.2	
Asian/Hawaiian Pacific Islander	0.5	1.7	4.7	
Other	5.1	5.6	9.4	
**Females (%)**	64.1	63.1	73.4	
**Restaurant Chain (%)**				
McDonalds	36.7	34.4	28.1	
Burger King	11.2	12.2	14.1	
Wendy's	31.1	36.7	46.9	
Kentucky Fried Chicken	20.9	16.7	10.9	

*p <0.10; **p <0.05; ***p <0.01;

Table 4 presents differences in food and beverage purchases and frequency of fast food consumption in 440 adults in NYC during the post-labeling period. Overall, 44.5% of adults reported not noticing the calorie information, 41% reported that they noticed the information but did not use it, and 14.5% reported that they both noticed and used the information. After controlling for covariates, the proportion of adults who purchased caloric beverages and salads was significantly lower among adults who noticed but reported not using the information compared to adults who reported not noticing the information. The frequency of fast food dinners and overall meals was also significantly lower among adults who noticed but reported not using the information compared to adults who reported not noticing the information. After controlling for covariates, the proportion of adults who ordered a salad was significantly greater among adults who noticed and reported using the information compared to adults who reported not noticing the information.

**Table 4:** Differences in Beverage and Food Consumption among Adults in NYC after Menu Labeling Legislation†

	Did Not See Calorie Information (Reference Group) (n = 196)	Saw Calorie Information But Did Not Use Information (n = 180)	Saw Calorie Information and Did Use Information (n = 64)
**Caloric Beverage (yes)**	50/196 = 26%	23/180 = 13%**	8/64 = 13%
**Ordered a Salad (yes)**	15/196 = 8%	9/180 = 5%**	12/64 = 19%**
**Type of Salad Dressing (regular)**	4/15 = 27%	3/9 = 33%	7/12 = 58%*
**Orders of French Fries**	67/196 = 34%	68/180 = 38%	26/64 = 41%
**Use of Cheese**	55/107 = 51%	63/110 = 57%	24/45 = 53%
**Dessert (yes)**	25/196 = 13%	28/180 = 16%	6/64 = 9%
**Mean Number of Fast Food Breakfasts per Week**	1.4	1.2	1.3
**Mean Number of Fast Food Lunches per Week**	2.5	2.3	1.9**
**Mean Number of Fast Food Dinners per Week**	1.3	1.0**	0.7**
**Mean Number of Snacks per Week**	1.4	1.0*	1.0
**Mean Number of Fast Food Meals per Week (including snacks)**	6.6	5.5**	4.9**

*p≤0.10; **p < 0.05; ***p < 0.01;

† Regression analysis controlling for age, race, gender, and restaurant type and standard errors were clustered at the restaurant level

Table 5 presents the magnitude of the difference in frequency of consuming fast food meals in adults in NYC during the post-labeling period. After adjusting for covariates, adults who noticed the labels but did not use the information consume 0.39 fewer fast food snacks and 1.12 fewer fast food meals (both p <0.05). Adults who noticed the labels and said they used the information consumed 0.64 fewer lunches and 0.48 fewer dinners compared to adults who did not notice the labels (both p <0.05).

**Table 5:** Magnitude of Differences in Frequency of Fast Food Consumption among Adults in NYC after Menu Labeling Legislation† (n = 435)

	Number of Fast Food Lunches per Week	Number of Fast Food Dinners per Week	Number of Snacks per Week	Number of Fast Food Meals per Week (including snacks)
**Saw Calorie Information But Did Not Use Information (95% CI) (n = 178)**	-0.25 (-0.59 - 0.09)	-0.28 (-0.50 - -0.06)**	-0.40 (-0.86 - 0.07)*	-1.12 (-1.90 - -0.35)***
**Saw Calorie Information and Did Use Information (95% CI) (n = 62)**	-0.64 (-1.27 - -0.10) **	-0.48 (-0.85 - -0.10)**	-0.34 (-0.84 - 0.15)	-1.38 (-2.92 - 0.15)*

*p≤0.10; **p <0.05; ***p <0.01;

† Regression analysis controlling for age, race, gender, and restaurant type. The reference group is adults who did not see calorie information. Standard errors were clustered based on restaurant.

Table 6 presents the predicted probabilities (holding all other variables at their means) for differences in food and beverage purchases in adults in NYC during the post-labeling period. After adjusting for covariates, an adult who noticed the calorie labels but did not use the information had a 13% chance of ordering a caloric beverage, which was significantly lower than an adult who did not notice the information (22%) (p <0.05). The probability of ordering a salad was 15% for an adult who noticed the labels and said they used the information, which was significantly greater than the probability (6%) for an adult who did not notice the information (p <0.05). The probability of ordering a salad was 4% for an adult who noticed the labels but did not use the information, which was significantly lower than the probability for an adult (6%) who reported not noticing the labels (p <0.05).

**Table 6:** Predicted Probabilities for Differences in Beverage and Food Consumption among Adults in NYC after Menu Labeling Legislation† (n = 435)

	Ordered a Caloric Beverage	Ordered a Salad	Ordered Regular Salad Dressing
**Did Not See Calorie Information (Reference Group) (n = 195)**	0.22	0.06	0.24
**Saw Calorie Information But Did Not Use Information (95% CI) (n = 178)**	0.13**	0.04**	0.39
**Saw Calorie Information and Used Information (95% CI) (n = 62)**	0.13	0.15**	0.57*

*p≤0.10; **p < 0.05; ***p < 0.01;

† Logistic Regression analysis and generated predicted probabilities controlling for age, race, gender, and restaurant type. Standard errors were clustered based on restaurant.

## Discussion

This study showed mixed findings. Although, a greater proportion of NYC adults ordered a caloric beverage and regular vs. low-fat salad dressing after calorie labeling compared to adults in Newark, NJ, calorie labeling may positively influence a subset of consumers and their food decisions. For example, adults who reported noticing and using calorie labels to inform their food choices consumed more salads and ate out at fast food restaurants less often than adults who did not notice the labels. Adults who noticed calorie labels but reported not using the information also ate at fast food restaurants less often and were less likely to order caloric beverages than adults who did not see the labels. This suggests that calorie labels may provide some benefit to all consumers who observe them, regardless of whether they report using them. Though these results are promising, it is not possible to definitively attribute these favorable differences to calorie labels, because adults who notice labels may differ from adults who do not notice labels. Adults who notice labels, for example, may have a stronger interest in health, which influences their food purchasing decisions.

The mixed findings of this analysis may be expected when evaluating a single public health intervention in the absence of other environmental changes. It is possible that the effects of calorie labeling have a larger influence on the overall population. For example, calorie labels may have encouraged some adults to stop frequenting fast food restaurants given the limited availability of healthful options. However, a study of Starbucks transactions in NYC, Boston, and Philadelphia found that, following implementation of calorie labels, the mean number of transactions at Starbucks increased but the mean revenue per transaction decreased somewhat, leading to an overall neutral effect of calorie labels on sales revenue [12].

In this study, a difference-in-difference analysis revealed some less favorable behavioral differences after calorie labeling in NYC, including a greater proportion of adults who ordered caloric beverages and regular salad dressing compared with adults in Newark, NJ. Given the limited availability of healthy alternatives in fast food restaurants, it is possible that some adults have reactionary responses to the labels and decide to make less healthy choices given the relatively small caloric benefit for making a more healthful decision - particularly if adults believe that the healthier options taste worse.

A recent study in Pierce County, Washington found that calorie labels voluntarily put on printed menus reporting the calorie, fat, sodium, and carbohydrate content of meals led to a significant reduction in the caloric and fat content of meals in approximately 20% of surveyed patrons [20]. Although the restaurant patrons reduced the caloric content of their meals by approximately 75 calories and fat content by 1.5 grams, more than 40% said that they noticed the information but did not make substantial changes to their food choices [20]. In another study, after calorie labeling, Starbucks patrons purchased 6% fewer calories on average (for a 12 calorie decrease), with most changes coming from lower calorie food items rather than beverages [12]. More substantial calorie reductions were noted in an experimental study [13] that manipulated printed restaurant menus to include calorie labels with and without educational information about recommended daily caloric intake. Adults consumed nearly 250 fewer calories during dinner and after the meal if they had menus with calorie labels and information about the recommended daily calorie intake compared to adults who had menus with only calorie labels. However, in another experimental study, this information was present and no impact of labeling was seen [7].

The results of this study, in combination with the relatively modest findings of similar studies, suggest a need for environmental strategies and social marketing or educational campaigns to maximize the efficacy of calorie labeling legislation. Public health interventions targeting behavioral change may work synergistically with other environmental strategies with similar objectives; for example, calorie labeling in combination with an increased presence of healthful food offerings in low-income neighborhoods and educational campaigns about total daily calorie requirements may lead to substantive behavioral change.

Research examining the most effective method for disseminating calorie information is also needed. For some menu items (e.g., burritos), it is allowable to post calorie ranges; often these ranges can spread more than 500 calories, making them difficult to interpret. Nutrition labeling on food packages has existed for some time now, yet a recent Health Canada survey notes that consumers still cite confusion interpreting labels as a barrier to their usage [21]. Further analysis may reveal that calorie labels must be both clear and specific to be maximally effective. Even labels that exclusively list calories are not necessarily very meaningful to consumers. Wisdom, Downs, and Lowenstein [22] presented consumers with a choice of multiple snacks and provided the calorie information in one of ten forms. In the numerical information conditions, consumers were presented with calorie information alone, calorie information with daily reference intakes, recommended calories for a snack (200), the percent daily snack calories, or the number of minutes on a treadmill it would require to burn the calories in the snack. Other consumers were presented with heuristic cues like a nutrition grading for each snack, a traffic light rating, or an expected body size for individuals who chose each snack. Overall, the heuristic cues, particularly the traffic light and expected body size conditions, were most effective though numerical information about calories did lead to some reductions in calorie intake. Treatment effects were stronger in overweight rather than normal weight consumers. Given recent evidence that most consumers are unaware of the number of daily calories needed to maintain their weight [23], it is more likely that calorie information will improve food choices when the information is easily translatable.

Beyond calorie labeling, it is important to consider the potential response of industry to this legislation. Calorie labels may encourage restaurants to formulate healthier tasty menu options to continue attracting a wide variety of consumers. Restaurants attempting to support consumer health may find that merely making it easier to choose more healthful items can increase the respective sales volume and revenue. A recent study suggests that changing defaults is more effective than pure informational approaches (i.e., listing more healthful versions of food items on menus requiring consumers to actively request a less healthful version of that option)[24]. This type of 'asymmetric paternalism' where consumers are encouraged to make healthful food choices while still having the autonomy to make less healthful food choices plays on known consumer biases seen in behavioral economics [25]. For example, consumers hold present-biased preferences, which means that they overvalue immediate rewards and costs compared with the rewards and costs of a delayed outcome [26,27]. Consequently, when healthier choices are easier to make, consumers exhibit a preference for the benefit of convenience. Moreover, when the healthy choice is the default option, consumers are more likely to make that choice even if a different, preferred option is available. This type of intervention has been useful with increasing retirement savings [28] and increasing organ donation rates [29]. Taken together, this suggests that calorie labeling has the potential to dramatically improve consumer well-being if labels are easy to interpret and if healthy choices become the default option.

There are a few important limitations to consider in the present study. First, this study was cross-sectional in design, and did not follow the same group of consumers over time. It is possible that the 4-week time period after legislation was enacted was insufficient to observe substantial behavioral change. However, the study of Starbucks transactions found that the small impact of calorie labeling in NYC was present immediately after labeling began [12]. Also, this study had low power due to a small sample size of adults in NYC after calorie labeling legislation. It is possible that greater behavioral differences would have been observed with three larger groups of adults who did or did not see or use the labels. Although the NYC calorie labeling legislation requires that calorie information be printed in the same size font as the price, it is unclear whether all restaurants in this analysis were compliant with this requirement.

Importantly, this study surveyed "real world" consumers rather than utilizing a laboratory setting, which may be more representative of normal life circumstance. This study was designed to gather data from low-income and minority populations in order to examine whether groups disproportionately affected by obesity and related health conditions are positively affected by calorie labels. Receipt data were collected by research assistants; the study did not use retrospective self-reports which are prone to greater error. To reduce variability by restaurant type, the same restaurants were surveyed before and after labeling. Because data were collected both before and after labeling in NYC and in a comparison city (Newark, NJ), all differences observed between pre- and post- labeling in NYC are more likely related to the passage of calorie labeling legislation rather than to other larger population trends.

## Conclusions

In this study, all adults who reported noticing calorie labels, regardless of whether they reported using it, made relatively healthier choices with respect to their fast food purchases than adults who did not notice calorie labels. Future research should focus on better understanding consumers' utilization of calorie labels including the factors that enhance their usage as well as the potential subconscious effects of calorie labeling. It is likely that calorie labeling legislation requires other environmental changes, social marketing, educational campaigns, industry change, and behavioral strategies to reach its maximum potential. However, nutrition and health professionals can use the existing calorie labels to assist consumers with making healthier food purchases by promoting their usage and publicizing recommended daily caloric intake. Nutrition and public health professionals can also advocate for consumers by encouraging restaurants to offer a wider array of healthful food and beverage choices.

## Competing interests

The authors declare that they have no competing interests.

## Authors' contributions

MV carried out the statistical analyses and drafted the manuscript. LBD was involved in conceiving the study and critically reviewing the draft for important intellectual content.

BE conceived of the study, participated in its design and coordination, provided statistical guidance, and critically reviewed the draft for important intellectual content. All authors read and approved the final manuscript.
